# Scutellarin Attenuates the IL-1β-Induced Inflammation in Mouse Chondrocytes and Prevents Osteoarthritic Progression

**DOI:** 10.3389/fphar.2020.00107

**Published:** 2020-02-26

**Authors:** Zucheng Luo, Zhichao Hu, Yujie Bian, Wenting Su, Xiaoyang Li, Shi Li, Jianbin Wu, Li Shi, Yonghuan Song, Gang Zheng, Wenfei Ni, Jixin Xue

**Affiliations:** ^1^ Department of Orthopaedics, The Second Affiliated Hospital and Yuying Children’s Hospital of Wenzhou Medical University, Wenzhou, China; ^2^ Department of Orthopaedics, Zhejiang Provincial Key Laboratory of Orthopaedics, Wenzhou, China; ^3^ The Second School of Medicine, Wenzhou Medical University, Wenzhou, China; ^4^ Department of Dermatology, Wenzhou Hospital of Integrated Traditional Chinese and Western Medicine, Wenzhou, China

**Keywords:** scutellarin, chondrocytes, osteoarthritis, inflammation, Nrf2/NF-κB

## Abstract

Osteoarthritis (OA) is a chronic degenerative disease wherein the articular cartilage exhibits inflammation and degradation. Scutellarin (SCU) is a flavonoid glycoside with a range of pharmacological activities, as shown in previous studies demonstrating its anti-inflammatory activity. How SCU impacts the progression of OA, however, has not been explored to date. Herein, we assessed the impact of SCU on murine chondrocytes in an OA model system. In *in vitro* assays, we measured chondrocyte expression of key OA-associated factors such as matrix metalloproteinase 13 (MMP-13), a disintegrin and metalloproteinase with thrombospondin motifs 5 (ADAMTS-5), cyclooxygenase-2 (COX-2), and inducible nitric oxide synthase (iNOS) *via* qRT-PCR and Western blotting, the expression of interleukin 6 (IL-6), tumor necrosis factor-α (TNF-α), and prostaglandin E2 (PGE2) were detected by qRT-PCR. Our results showed that the downregulation of MMP-13, ADAMTS-5, COX-2, and iNOS expression by SCU and the overproduction of IL-6, TNF-α, and PGE2 induced by IL-1β were all inhibited by SCU in a concentration-dependent manner. Moreover, SCU was able to reverse aggrecan and collagen II degradation and nuclear factor-κB (NF-κB) and nuclear factor erythroid-derived 2-like 2 (Nrf2) signaling pathway activation both *in vivo* and *in vitro*. We further used a destabilization of the medial meniscus (DMM) murine model of OA to explore the therapeutic benefits of SCU *in vivo*. Together, our findings suggest SCU to be a potentially valuable therapeutic agent useful for treating OA.

## Introduction

Osteoarthritis (OA) is a progressive disease wherein the articular cartilage undergoes chronic and progressive destruction (Loeser, 2009; Khan et al., 2017). Pathological findings in OA patients include chondrocyte hypertrophic differentiation, synovial inflammation, osteophyte formation, and subchondral bone remodeling in addition to this loss of the articular cartilage (Heinegard and Saxne, 2011; Glyn-Jones et al., 2015). A number of factors associated with OA risk have been identified, including age, sex, body mass index (BMI), a history of joint injuries, and joint shape/alignment, but the exact mechanisms underlying the pathogenesis of this disease remain poorly defined (Prieto-Alhambra et al., 2014; Loeser et al., 2016). Indeed, a number of factors are likely to influence the structural and functional alterations that characterize OA, including oxidative stress, impaired mitochondrial activity, expression of catabolic genes, and altered chondrocyte inflammatory activity (Goldring, 2000; Loeser et al., 2012). Inflammatory mediators have been found to be key mediators of OA pathology (Sokolove and Lepus, 2013), with the proinflammatory cytokine interleukin-1β (IL-1β) having been shown to drive inflammation, oxidative stress, and chondrocyte apoptosis in this disease (Haseeb and Haqqi, 2013). The inflammation-regulating transcription factor nuclear factor-κB (NF-κB) is also essential to this IL-1β-mediated induction of inflammatory and catabolic genes in chondrocytes (Kumar et al., 2004). Meanwhile, NF-κB signaling pathways could be activated by a wide range of stimuli, such as tumor necrosis factor-α (TNF-α) and IL-1β. By signaling through NF-κB, IL-1β can induce a disintegrin and metalloproteinase with thrombospondin motifs 5 (ADAMTS5) expression, in addition to upregulation of matrix metalloproteinases (MMPs) that can break down cartilaginous tissue. In this context, inhibition of NF-κB activation and transcription is an effective way by which OA exerts its anti-inflammatory effect. It has been demonstrated that nuclear factor erythroid-derived 2-like 2 (Nrf2)/heme oxygenase-1 (HO-1) pathway is involving in inflammation disease (Feng et al., 2018; Hu et al., 2019). Moreover, Nrf2 signaling has been shown to be a key anti-inflammatory defense mechanism that enhances expression of HO-1 and inhibits NF-κB activity, which could decrease the generation of inflammatory mediators such as MMPs and PGE2 and attenuate the extracellular matrix (ECM) degradation, making this a potentially viable therapeutic target in OA (Chen et al., 2006).

**Figure 1:**
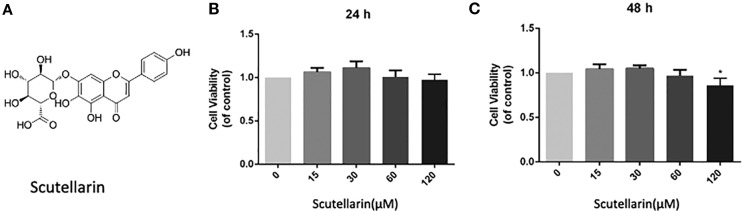
Scutellarin (SCU) affects chondrocyte viability. SCU chemical structure **(A)**. The impact of SCU on chondrocyte viability across a range of concentrations was assessed *via* CCK-8 assay after 24 **(B)** and 48 h **(C)**. Data are means ± SD. *P < 0.05 vs. control; n = 5.

Scutellarin (SCU) (4′,5,6-trihydroxy flavonoid-7-glucuronide; **Figure 1A**) is a flavonoid glycoside isolated from *Erigeron breviscapus* (Zhang et al., 2009). We have previously shown SCU to exhibit potent biological activity, with anti-inflammatory (Wang et al., 2011), anti-oxidative (Hong and Liu, 2007), and anti-apoptotic activity (Dai et al., 2011). Importantly, SCU is able to induce Nrf2/HO-1 activity and to inhibit NF-κB signaling in hepatocytes (Zhang et al., 2018), osteoblasts (Wang et al., 2018b), and microglial cells (Yuan et al., 2015). However, the study of the value of SCU as an inhibitor of inflammation and oxidative stress in the context of OA remains limited. Therefore, in the present report, we assessed the ability of SCU to protect against inflammation and ECM breakdown in response to IL-1β treatment. We then extended these studies into a mouse model of OA to explore the therapeutic value of this compound.

## Materials and methods

### Reagents

SCU (⩾98% pure) was from Solarbio (Beijing, China), while DMOS and type II collagenase came from Sigma-Aldrich (MO, USA). Antibodies specific for aggrecan, collagen II, MMP-13, HO-1, ADAMTS5, β-actin, inducible nitric oxide synthase (iNOS), and Lamin B1 came from Abcam (Cambridge, UK), as was AF488-labeled goat anti-rabbit IgG. Anti-Nrf2 and secondary goat anti-rabbit immunoglobulin G (IgG) were from Bioworld (OH, USA), while anti-cyclooxygenase-2 (COX-2) was from Cell Signaling Technology (MA, USA). Recombinant IL-1β came from PeproTech (NJ, USA). 4′,6-Diamidino-2-phenylindole (DAPI) was from Beyotime (Shanghai, China).

### Primary Mouse Chondrocyte Culture

We euthanized 2-week-old C57BL/6 mice and used these animals to isolate primary chondrocytes as in previous reports (Zheng et al., 2018). Briefly, we isolated cartilage from the knee joints of these animals prior to treating them for 4–6 h using 2 mg/ml (0.2%) collagenase II at 37°C. We then spun down digests for 5 min at 1,000 rpm, discarded the supernatants, resuspended the cells, and plated them prior to incubation at 37°C in a humidified 5% CO_2_ incubator. After growing to 80%–90% confluence, cells were harvested with 0.25% trypsin–EDTA solution. Cells from the second passage were used for all experimental studies.

### Animal Model

The Animal Care and Use Committee of Wenzhou Medical University approved this study (ethics code: wydw2017-0159). We obtained C57BL/6 wild-type (WT) mice from the Animal Center of the Chinese Academy of Sciences (Shanghai, China). Consistent with past studies (Glasson et al., 2007), we induced OA in these animals *via* surgically destabilizing the medial meniscus (DMM). For this approach, animals were anesthetized before cutting into the joint capsule medial to the patellar tendon, followed by transection of the medial meniscotibial ligament. After animals had recovered from surgery, they were randomized into sham, vehicle control, and SCU-treated groups.

### Experimental Design

To explore SCU anti-inflammatory, chondrocytes were treated for 24 h with IL-1β (10 ng/ml) following pretreatment with SCU (0, 15, 30, or 60 μM).

To assess the therapeutic value of SCU *in vivo*, we used a murine DMM model as outlined above, with mice in the SCU treatment group receiving a once daily intraperitoneal (i.p.) SCU injection (50 mg/kg/day) for 8 weeks. Vehicle control animals were instead injected with saline. At the eighth postoperative week, animals were euthanized, and samples of cartilage tissue were isolated.

### CCK-8 Assay

A CCK-8 kit (Dojindo Co., Kumamoto, Japan) was utilized in order to gauge the impact of SCU on chondrocyte viability based on provided directions. Initially, chondrocytes were plated into 96-well plates (6 × 10³ cell/cm^2^) for 12 h, after which SCU (15, 30, 60, or 120 μM) was added for 24 or 48 h. After washing in PBS, 10 μl/well of CCK-8 solution was added for 4 h at 37°C. Absorbance in each well at 450 nm was then assessed *via* microplate reader (Model 550, Bio-Rad, USA). Triplicate experiments were performed.

### Enzyme-Linked Immunosorbent Assay

ELISA kits (R&D Systems, MN, USA) were used based on provided directions.

### Immunofluorescence Analysis

Chondrocytes (4 × 10^5^ cells/ml) were added to glass six-well plates for 24 h, after which they were washed in PBS, fixed for 15 min using 4% paraformaldehyde, permeabilized for 15 min using 0.4% Triton X-100, blocked for 1 h with 10% goat serum, rinsed, and then probed overnight at 4°C with antibodies recognizing collagen II (1:200), p65 (1:200), and Nrf2 (1:100). After additional washing with PBS, cells were then stained using an appropriate fluorescein isothiocyanate (FITC)-labeled secondary antibody (1:400) for 1 h, after which DAPI was added for 2 min to achieve nuclear staining. A fluorescent microscope (Olympus) was then used to visualize samples.

### qRT-PCR

TRIzol (Invitrogen) was used to isolate RNA from treated chondrocytes based on provided directions. Next, 1 μg of RNA from each sample was used to prepare cDNA *via* reverse transcription (MBI Fermentas, Germany). Thermocycler settings were 95°C for 10 min, then 40 cycles of 95°C for 15 s, 60°C for 1 min. A CFX96Real-Time PCR System (Bio-Rad, CA, USA) was used for all reactions. Glyceraldehyde 3-phosphate dehydrogenase (GAPDH) expression was used to normalize all Ct values, and the 2^-ΔΔCt^ approach was used to calculate relative gene expression. The NCBI Primer-Blast Tool was used to design specific primers, which are listed in **Table 1**.

**Table 1:** Primers used in this study.

Gene	Forward primer	Reverse primer
COX-2	5′-TCCTCACATCCCTGAGAACC-3′	5′-GTCGCACACTCTGTTGTGCT-3′
iNOS	5′-GACGAGACGGATAGGCAGAG-3′	5′-CACATGCAAGGAAGGGAACT-3′
IL-6	5′-CCGGAGAGGAGACTTCACAG-3′	5′-TCCACGATTTCCCAGAGAAC-3′
TNF-α	5′-ACGGCATGGATCTCAAAGAC-3′	5′-GTGGGTGAGGAGCACGTAGT-3′
COX-1	5′-CTTTGCACAACACTTCACCCACC-3′	5′-AGCAACCCAAACACCTCCTGG-3′
COX-2	5′-GCATTCTTTGCCCAGCACTT-3′	5′-AGACCAGGCACCGACCAAAGA -3′
mPGES-1	5′-CTGCTGGTCATCAAGATGTACG-3′	5′-CCCAGGTAGGCCACGTGTGT-3′
mPGES-2	5′-AAGACATGTCCCTTCTGC -3′	5′-CCAAGATGGGCACTTTCC-3′

COX, cyclooxygenase; iNOS, inducible nitric oxide synthase; IL-6, interleukin 6; TNF-α, tumor necrosis factor α; mPGES-2, microsomal prostaglandin E synthase 2.

### Western Blot Assay

Radioimmunoprecipitation assay (RIPA) buffer containing 1 mM phenylmethylsulfonyl fluoride (PMSF) was used to lyse samples, which were then spun at 12,000 rpm for 15 min at 4°C. Supernatant protein levels were then assessed *via* bicinchoninic acid (BCA) assay (Beyotime), and 40 ng protein from each sample was separated *via* sodium dodecyl sulfate (SDS)-polyacrylamide gel electrophoresis (PAGE) and transferred to polyvinylidene fluoride (PVDF) membranes (Bio-Rad, USA) that were blocked for 2 h using 5% nonfat milk, after which they were probed overnight using primary antibodies specific for aggrecan, collagen II, β-actin, Nrf2 (1:500), HO-1 (1:5,000), COX-2, iNOS, MMP-13, ADAMTS-5, p65, IκBα, and Lamin B1 at 4°C. Antibodies were used at a 1:1,000 dilution unless otherwise indicated. After washing, blots were then probed for 2 h with appropriate secondary antibodies were visualized *via* enhanced chemiluminescence (Invitrogen), with Image Lab 3.0 (Bio-Rad) used to quantify band density.

### Molecular Modeling

Nrf2 (PDB ID: 4ZY3) was subjected to docking studies (Khan et al., 2017). The protein was downloaded from PDB (https://www.rcsb.org/) before being prepared for docking. The lowest energy conformations for docking were determined *via* default parameters after being minimized using PyMoL (version 1.7.6). The protein–ligand docking analysis was carried out using AutoDockTools (version 1.5.6), which can provide ligand binding flexibility with the binding pocket residues. Finally, the images were generated using UCSF PyMoL.

### X-Ray Imaging Method

The animals were subjected to X-ray examination at 8 weeks postoperatively with or without treatment. X-ray imaging was performed for all mice to assess osteophyte formation, joint spaces, and calcification changes of the cartilage surface using a digital X-ray machine (Kubtec Model XPERT.8; KUB Technologies Inc.).

### Histopathologic Analysis

After fixation for 24 h in 4% paraformaldehyde (PFA), knee joints (n = 15/group) were decalcified for 2 months in 10% ethylenediaminetetraacetic acid (EDTA). Samples then underwent paraffin embedding and were prepared as 5-μm-thick sections. Safranin O-fast green (SO) was then used to stain each cartilage sample. Cartilage and subchondral bone cellularity and morphology were then assessed using the Osteoarthritis Research Society International (OARSI) scoring system in a blinded fashion for the medial femoral condyle and the medial tibial plateau, as in past reports (Glasson et al., 2010).

### Statistical Analysis

Data are means ± standard deviations (SDs). SPSS 20.0 was used for all analyses. One-way ANOVAs with Tukey’s test were used to compare control and treatment group data, while nonparametric results were compared *via* the Kruskal–Wallis H test. p < 0.05 was the significance threshold.

## Results

### SCU Impact on the Viability of Chondrocytes

We began by analyzing the cytotoxicity of SCU (**Figure 1A**) when used to treat chondrocytes for 24–48 h with 0, 15, 30, 60, or 120 μM of SCU *via* a CCK-8 assay. We found that optimal chondrocyte viability was achieved when cells were treated using 60 μM SCU (**Figures 1B, C**). As such, doses of 15, 30, or 60 μM SCU were used in subsequent experiments.

### SCU Protects Chondrocytes Against IL-1β-Mediated Induction of Inflammatory Mediators

**Figure 2:**
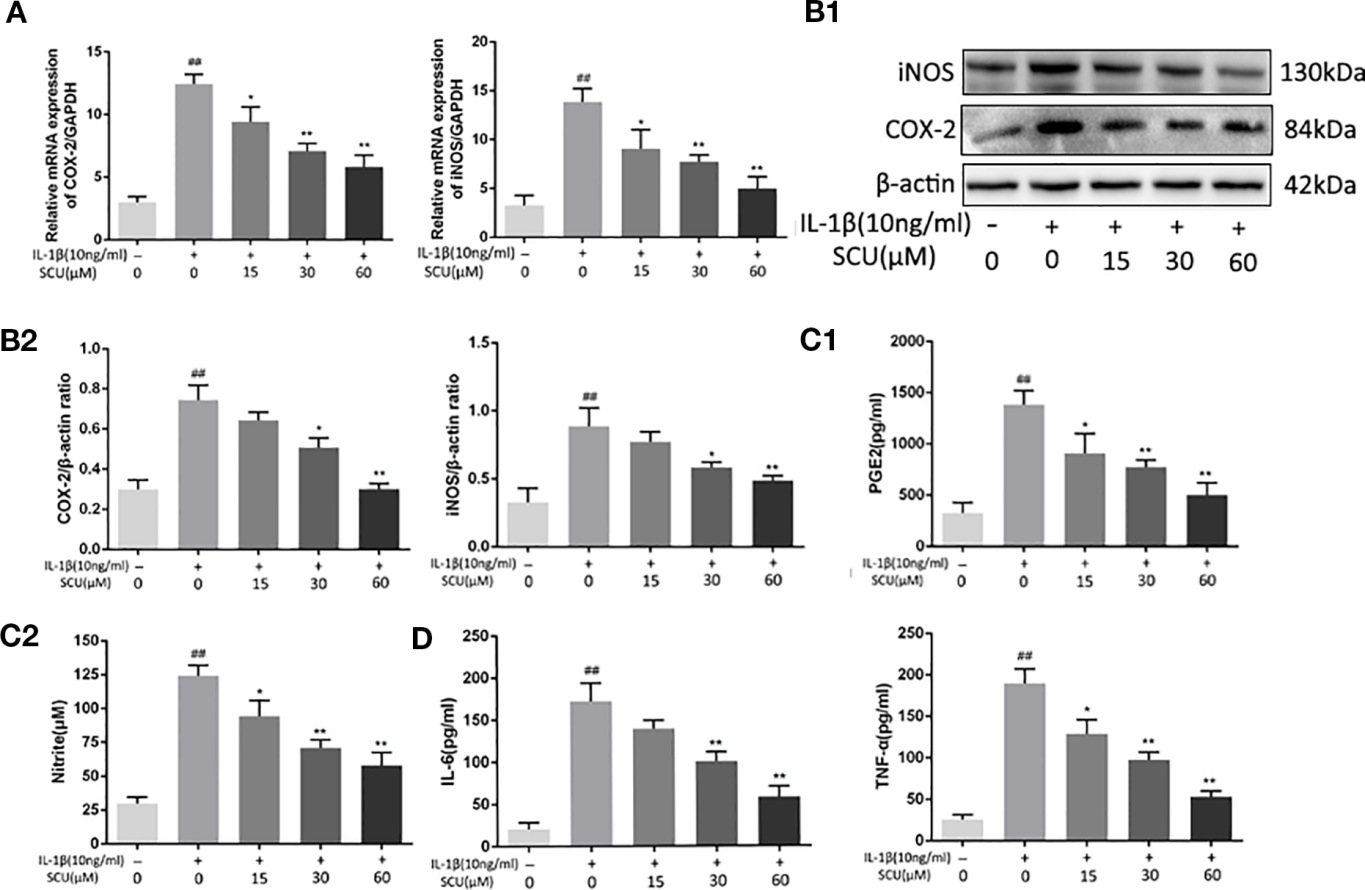
Scutellarin (SCU) inhibits chondrocyte inflammation. Expression of cyclooxygenase-2 (COX-2) and inducible nitric oxide synthase (iNOS) were measured *via* qRT-PCR **(A)**. Chondrocyte COX-2 and iNOS protein levels were measured *via* Western blotting **(B)**. ELISAs were used to assess levels of prostaglandin E2 (PGE2), tumor necrosis factor-α (TNF-α), and interleukin 6 (IL-6) **(C**, **D)**. Data are means ± SD. ^##^P < 0.01 vs. control; *P < 0.05, **P < 0.01 vs. IL-1β-only; n = 5.

We next explored the impact of SCU on chondrocyte inflammation in response to IL-1β (10 ng/ml) by treating these cells with the indicated SCU doses for 24 h before IL-1β addition. We then assessed the expression of key inflammatory mediators. We found that upon treatment using 15 μM SCU prior to IL-1β treatment, this did not significantly alter subsequent COX-2 (p = 0.1148) and iNOS (p = 0.2714) protein levels relative to control, but SCU markedly reduced IL-1β-mediated increases in iNOS and COX-2 expression at 30 μM SCU (**Figures 2A, B**), and that it modulated the production of endogenous NO and PGE2 in a dose-dependent manner (**Figure 2C**). IL-1β induced increased NO and PGE2 production, whereas SCU decreased the production of these factors in a dose-dependent fashion. SCU was also able to dose-dependently inhibit IL-β-induced secretion of TNF-α and IL-6 by chondrocytes (**Figure 2D**), although this difference was not significant for the 15 μM SCU dose with respect to IL-6 production (p = 0.0745). Together, these findings suggested that SCU was able to prevent upregulation of inflammatory mediators in chondrocytes upon IL-1β treatment in a dose-dependent fashion.

### SCU Protects Against IL-1β-Mediated Chondrocyte ECM Degradation

**Figure 3:**
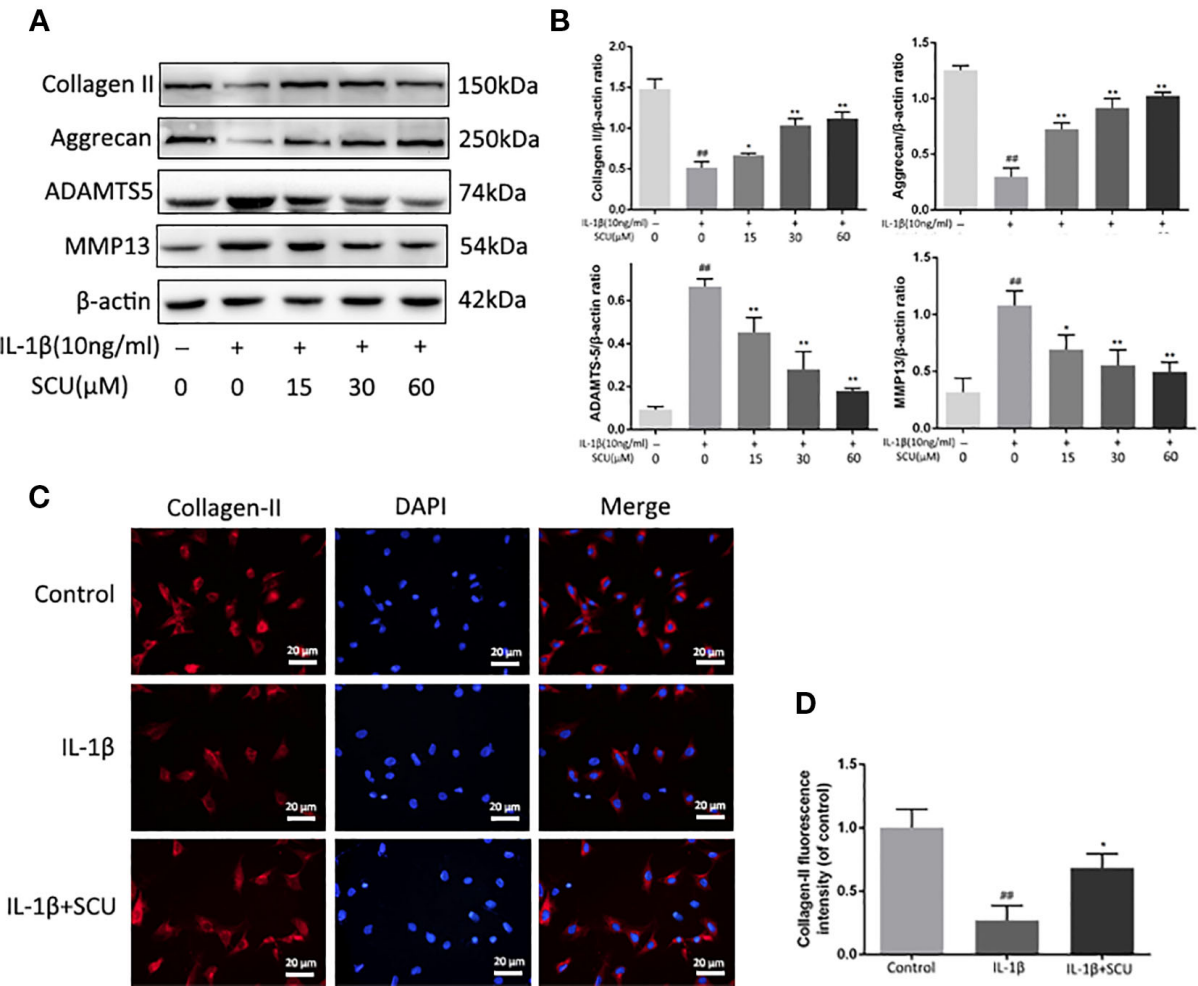
Scutellarin (SCU) inhibits murine chondrocyte extracellular matrix (ECM) degradation. Levels of aggrecan, matrix metalloproteinase 13 (MMP-13), collagen II, and a disintegrin and metalloproteinase with thrombospondin motifs 5 (ADAMTS-5) in the supernatants collected from chondrocytes treated as above **(A**, **B)**. Collagen II **(C)** was analyzed *via* immunofluorescent staining, with 4′,6-diamidino-2-phenylindole (DAPI) used for nuclear detection (scale bar = 20 μm). Collagen-II fluorescent intensity was quantified using ImageJ **(D)**. Data are means ± SD. ^##^P < 0.01 vs. control; *P < 0.05, **P < 0.01 vs. IL-1β-only; n = 5.

We next explored the impact of SCU treatment on the IL-1β-induced degradation of the ECM *via* measuring aggrecan, collagen-II, ADAMTS5, and MMP-13 levels in these chondrocytes *via* Western blotting. We found that IL-1β treatment led to a marked reduction in aggrecan and collagen II levels, with corresponding increases in MMP-13 and ADAMTS5 levels (**Figures 3A, B**). Pretreatment with SCU reversed this phenotype in a dose-dependent manner. Furthermore, immunofluorescent staining of collagen II exhibited a comparable phenotype to that observed by Western blotting (**Figures 3C, D**).

### SCU Suppressed NF-κB Activation in Chondrocytes Upon IL-1β Treatment

**Figure 4:**
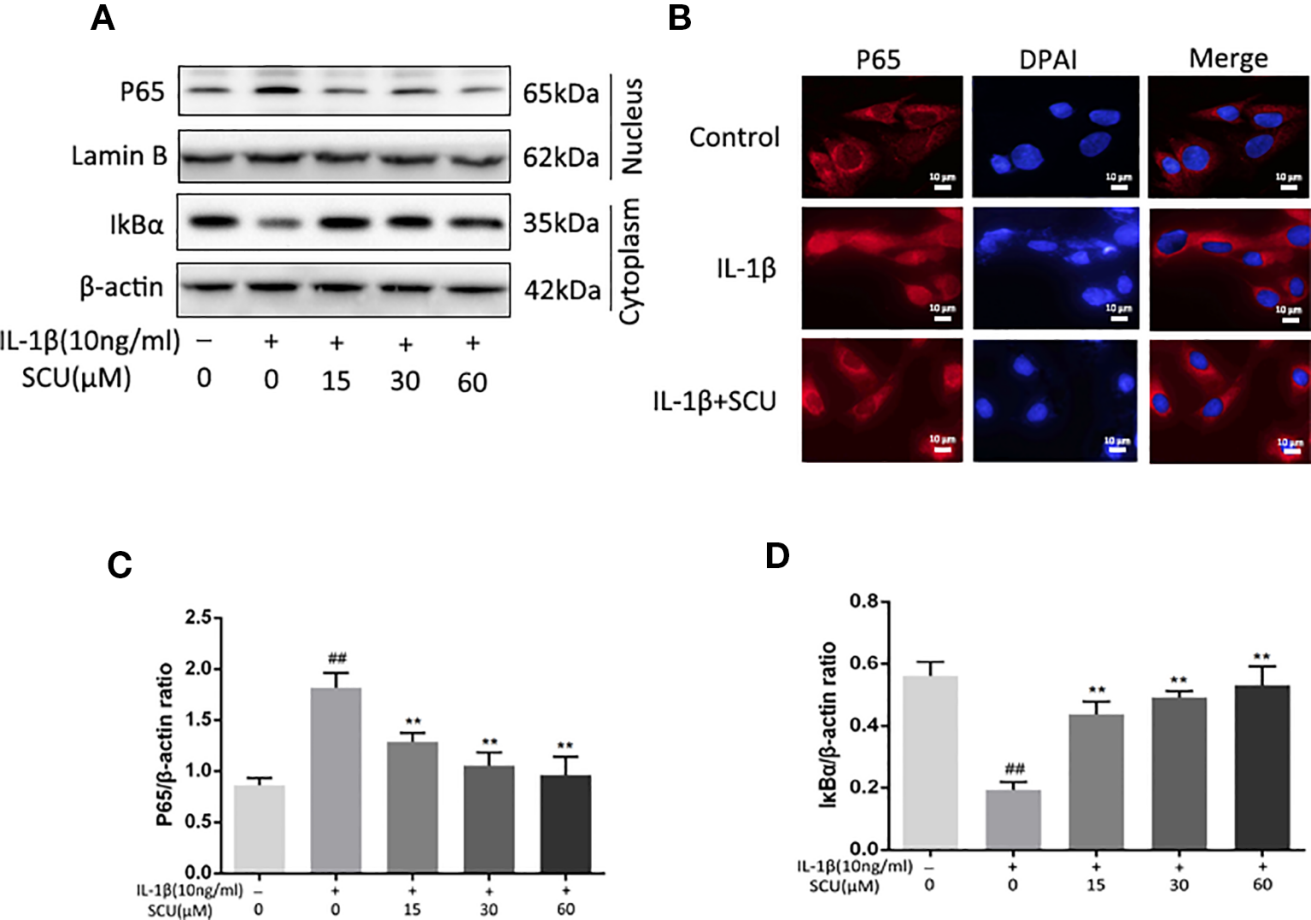
Scutellarin (SCU) affects interleukin (IL)-1β-induced nuclear factor (NF)-κB activation. Cytoplasmic IκBα and nuclear p65 levels in chondrocytes were assessed *via* Western blotting **(A)** and were quantified **(C**, **D)**. **(B)** p65 nuclear translocation was assessed *via* immunofluorescent staining, with 4′,6-diamidino-2-phenylindole (DAPI) used for nuclear detection (scale bar: 10 μm). Data are means ± SD. ^##^P < 0.01 vs. control; **P < 0.01 vs. IL-1β-only; n = 5.

Next, we assessed the impact of SCU on activation of NF-κB signaling *via* measuring cytoplasmic IκBα and nuclear p65 protein levels in chondrocytes following IL-1β stimulation. IL-1β led to marked degradation of IκBα and p65 nuclear translocation within 2 h of treatment (**Figures 4A, C**, and **D**). Pretreatment with SCU, however, inhibited this effect in a dose-dependent fashion. Consistent with this, immunofluorescent analyses revealed that IL-1β treatment was sufficient to induce rapid nuclear localization of p65 relative to control cells, while pretreatment of cells with 60 μM SCU was sufficient to prevent this effect (**Figure 4B**). Together, these findings suggested that SCU was able to reduce the IL-1β-mediated activation of chondrocyte NF-κB signaling.

### SCU Regulates Chondrocyte Nrf2/HO-1 Signaling

**Figure 5:**
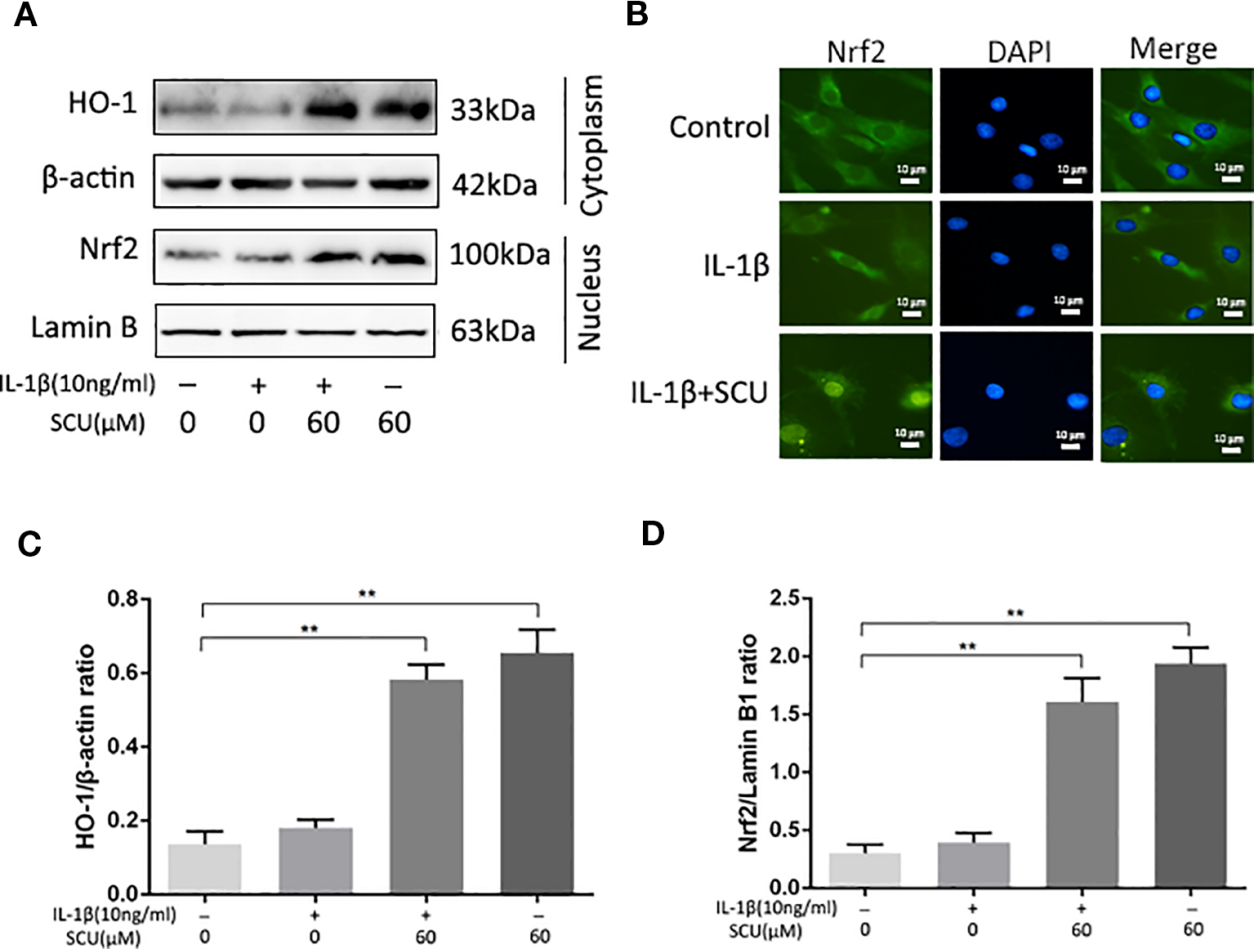
Scutellarin (SCU) affects the nuclear factor erythroid-derived 2-like 2 (Nrf2)/heme oxygenase-1 (HO-1) pathway. Nuclear Nrf2 and cytoplasmic HO-1 levels in chondrocytes were assessed *via* Western blotting **(A)** and were quantified **(C**, **D)**. **(B)** Nrf2 nuclear translocation was assessed *via* immunofluorescent staining, with 4′,6-diamidino-2-phenylindole (DAPI) used for nuclear detection (scale bar: 10 μm). Data are means ± SD. **P < 0.01 vs. control; n = 5.

Previous work suggests that SCU can mediate anti-inflammatory activity in part *via* activation of the Nrf2/HO-1 pathway (Guo et al., 2014). We therefore measured Nrf2 and HO-1 levels in IL-1β-treated chondrocytes *via* Western blotting, revealing a significant increase in Nrf2 nuclear translocation and HO-1 expression in cells that had been treated with SCU ± IL-1β treatment (**Figures 5A, C, D**). Immunofluorescent staining results were consistent with these Western blotting findings (**Figure 5B**). Together, these data indicated that pretreatment with SCU was sufficient to promote Nrf2 nuclear translocation and HO-1 expression, thereby mediating anti-inflammatory activity.

### Molecular Docking

**Figure 6:**
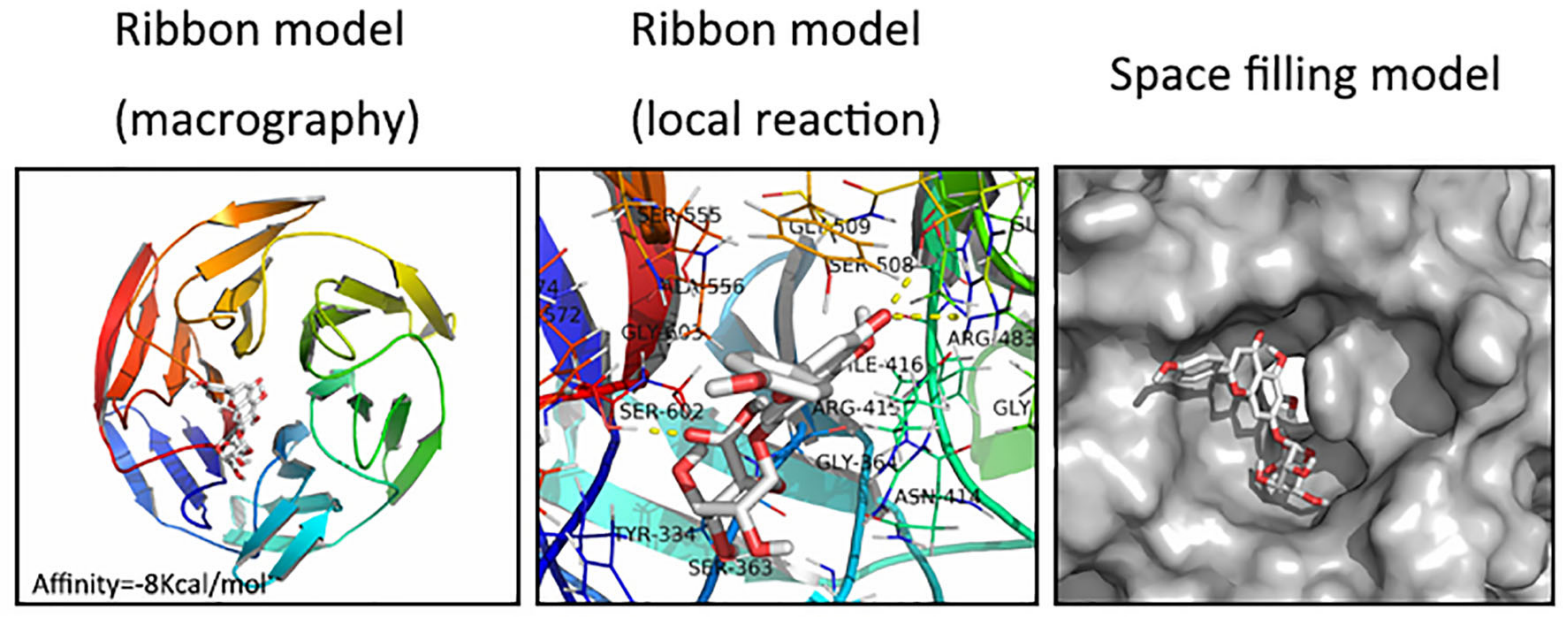
Scutellarin (SCU) interacts with nuclear factor erythroid-derived 2-like 2 (Nrf2) in a docking study. A ribbon model is used to represent protein residues, with a two-dimensional (2D) binding model shown. SCU was able to dock strongly within the Nrf2 binding site (affinity = -8 kcal/mol). This proposed binding interaction involves SCU interacting with SER508, SER602, and ARG483 on Nrf2. The binding of SCU in the Nrf2 pocket is shown using a space filling model.

We next assessed whether there was any affinity between SCU and Nrf2 or upstream proteins in the Nrf2 pathway *via* a computational molecular docking analysis (Khan et al., 2017). For this analysis, we utilized the SCU chemical structure shown in **Figure 1A**. After examination of all generated models, we found SCU to clearly interact with and dock in the Nrf2 binding site (**Figure 6**), with macro- and local-level views of these interactions shown using a ribbon model. We additionally utilized a space-filling model to illustrate this interaction. We observed high-affinity (-8 kcal/mol) hydrogen binding events between SCU and the SER508, SER602, and ARG483 residues of Nrf2. This suggested that SCU may function to inhibit the development of OA in part through its ability to interact with Nrf2 in a manner that promotes its nuclear translocation.

### SCU Efficacy in a Murine Model of OA

**Figure 7:**
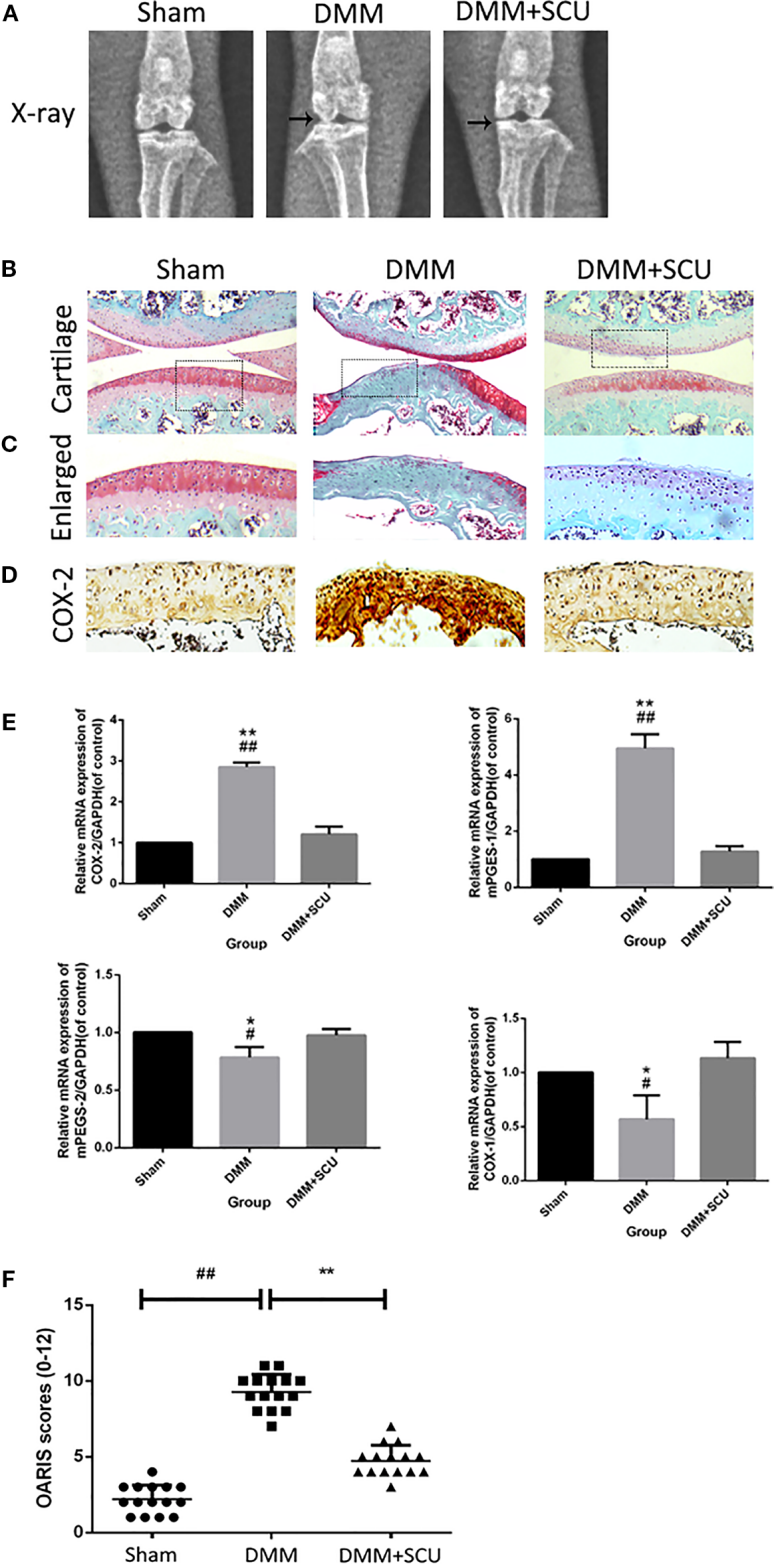
Scutellarin (SCU) slows osteoarthritis (OA) development in a murine destabilization of the medial meniscus (DMM) model system. OA progression was gauged based on X-ray assessment of murine knee joints, with joint space narrowing being evident in the OA and treated groups, and with clear signs of cartilage calcification in the OA group (black arrows) **(A)**. Representative cartilage SO staining at 8 weeks after operation (scale bar: 200 μm) **(B**, **C)**. Immunohistochemical analysis of COX-2 **(D)**. Expression of cyclooxygenase (COX)-1, COX-2, microsomal prostaglandin E synthase 1 (mPGES-1), and mPGES-2 were measured *via* qRT-PCR **(E)**. Cartilage OARSI scores **(F)**. Data are means ± SD. ^#^P < 0.05, ^##^P < 0.01 vs. sham; *P < 0.05, **P < 0.01 vs. DMM + SCU group; n = 15.

In a final set of experiments, we used a murine DMM model of OA to assess the therapeutic effect of daily administration of SCU (50 mg/kg, intraperitoneally) over an 8-week period. We assessed morphological and histological changes in mice *via* X-ray analysis and SO staining. Relative to sham control mice, OA model mice exhibited clear joint space narrowing and increased cartilage surface density, whereas these effects were reduced somewhat in SCU-treated animals (**Figure 7A**). SO staining (**Figures 7B, C**) further revealed that while articular cartilage in sham control animals was smooth and stained red (positive), animals in the OA group exhibited clear signs of cartilage abrasion, proteoglycan loss, and hypocellularity relative to these controls. Notably, animals treated with SCU exhibited a smoother cartilage surface and reduced loss of proteoglycan. For both COX and microsomal PGE synthase (mPGES), COX-1 and mPGES-2 were suppressed expressed whereas COX-2 and mPEFS-1 were inducible in the OA group; fortunately, the opposite was true in the SCU-treated group (**Figures 7D, E**). Furthermore, SCU treatment was associated with a reduction in OARSI scores relative to OA model animals (**Figure 7F**). As such, these findings indicate that SCU is able to significantly reduce the rate and/or severity of OA progression *via* impairing cartilage destruction, calcification, and osteophyte formation *in vivo*.

## Discussion

OA is the most common form of chronic joint disease, having a profound and negative impact on the quality of life for those affected by this condition (Van Den Bosch, 2018). Over 237 million individuals globally suffer from OA, with rates being highest among older adults. In addition to being a chronic degenerative disease, inflammation has been shown to be a key mediator of OA (Cicuttini and Wluka, 2014; Pers et al., 2015). Currently, OA disease management largely relies upon the utilization of nonsteroidal anti-inflammatory drugs (NSAIDs) (Sasaki et al., 1998; Sun et al., 2017); and no effective compounds capable of reliably curing or altering the course of this disease have been found. There is thus an urgent need to identify better treatment options of patients affected by OA.

SCU is an herbal derived from *E. breviscapus* (Vant.) that is often used in Chinese medicine, exhibiting a range of pharmacological and clinical activities (Wang and Ma, 2018). SCU has previously been used successfully to cardiovascular and cerebrovascular diseases (Wang et al., 2016; Wang and Ma, 2018), and it has exhibited anti-apoptotic, anti-inflammatory, and anticancer efficacy in preclinical research (Dai et al., 2011; Du et al., 2018; Nie et al., 2018). At present, however, the efficacy of SCU as a means of treating OA has not been well-studied. In the present report, we found that SCU was able to protect against IL-1β-mediated chondrocyte inflammation and ECM dysregulation *via* activation of Nrf2/HO-1 signaling and inhibition of NF-κB signaling.

The NF-κB pathway is well-documented to be a key regulator of OA pathology and progression (Hou et al., 2017; Lepetsos et al., 2018). When cells respond to IL-1β, this leads to the phosphorylation of IκB in the cytosol, leading in turn to the phosphorylation of the NF-κB p65 subunit. This subunit then translocates to the nucleus and facilitates the enhanced expression of pro-inflammatory and catabolic genes (Roman-Blas and Jimenez, 2006). In these same IL-1β-stimulated chondrocytes, iNOS catalyzes NO production, leading to increased MMP expression and reduced collagen II and proteoglycan generation and thereby resulting in the degradation of the ECM (Arasapam et al., 2006). COX-2 mediates the production of inflammatory PGE2 from arachidonic acid, leading to additional MMP- and ADAMTS5-mediated degradation of the ECM (Hardy et al., 2002). These MMPs, and particularly MMP-13, are key mediators of collagen II degradation, while the ADAMTS proteins target aggrecan for enzymatic breakdown, with ADAMTS5 being the most prominent member of this protein family. Prolonged MMP and ADAMT protein expression can lead to complete destruction of the articular ECM and the structure of the affected joint, resulting in permanent disability (Verma and Dalal, 2011; Kang et al., 2017). This thus suggests that therapeutic compounds capable of inhibiting the expression and/or activity of MMP-13 and ADAMTS5 may represent ideal agents for treating OA. In the present study, we found that SCU was able to suppress NO, PGE2, IL-6, and TNF-α production as well as COX-2 and iNOS upregulation at the RNA and protein levels. SCU further inhibited MMP-13 and ADAMT5 and the breakdown of aggrecan and collagen II in OA-model chondrocytes. This work thus suggests that SCU is able to effectively suppress inflammation induced by IL-1β in the context of OA owing to its ability to suppress NF-κB pathway activation.

Previous work has shown that the ability of SCU to inhibit NF-κB activity is linked to the AKT/NF-κB, p38/c-Jun N-terminal kinase (JNK), and NF-κB/mitogen-activated protein kinase (MAPK) pathways (Zhang et al., 2017). However, Nrf2/HO-1 signaling is also a key anti-oxidative stress response pathway in cells (Wang et al., 2018a), with Nrf2 targeting HO-1, which in turn mediates anti-inflammatory (Tenhunen et al., 1968; Kim et al., 2007; Hsu et al., 2014). Nrf2/NF-κB cross talk is also known to be a key pathway regulating downstream protein expression (Liu et al., 2008; Lee et al., 2009). The interplay between the Nrf2 and NF-κB-mediated inﬂammatory responses is a complex process. Inhibition of Nrf2 is associated with enhanced inﬂammation while its activation decreases pro-inﬂammatory responses transcriptionally regulated by NF-κB (Sivandzade et al., 2019). We found that SCU was able to enhance Nrf2-dependent expression of HO-1 and to thereby suppress NF-κB activity (**Figure 5**). We further found that SCU was able to directly interact with certain residues in the Nrf2 binding pocket with high-affinity through a docking analysis. SCU thereby led to enhanced HO-1 expression and suppressed p65 phosphorylation. While there may be other mechanisms that regulate the degree to which this mechanism functions in all contexts, our results strongly suggest that SCU is able to reduce ECM degradation in OA at least in part through the Nrf2/HO-1 pathway.

Furthermore, SCU directly occupied the binding pocket of Nrf2 by interacting with some amino acid residues, and the affinity was fairly high in the docking analysis results. Consequently, SCU increased the expression of HO-1 and suppressed the phosphorylation of P65. Although a potential upstream or bypass mechanism might exist, our data suggested that SCU attenuated ECM degradation by the Nrf2/HO-1 signal pathway.

**Figure 8:**
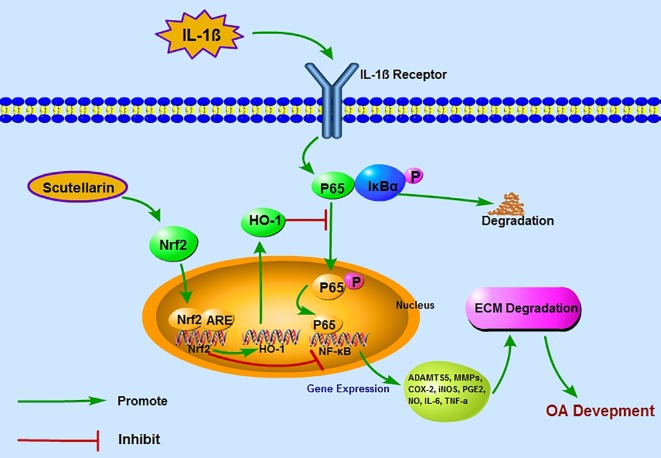
A proposed model for the protective role of scutellarin (SCU) in osteoarthritis (OA), wherein it suppresses nuclear factor (NF)-κB activity *via* the nuclear factor erythroid-derived 2-like 2 (Nrf2)/heme oxygenase-1 (HO-1) pathway. Red and green arrows correspond to negative and positive regulatory effects, respectively.

In summary, we have provided clear evidence that SCU is able to markedly constrain the upregulation of inflammatory and catabolic genes in response to IL-1β stimulation owing to its ability to inhibit NF-κB activity to activate Nrf2-dependent expression of HO-1 in murine OA model chondrocytes (**Figure 8**). We further found SCU to have a high affinity for the Nrf2 binding pocket, consistent with these *in vitro* findings. Importantly, *in vivo* SCU was able to protect against ECM degradation and chondrocyte loss in a murine model of OA, suggesting that this compound may be a potentially viable treatment for this disorder. While further work is needed to validate and refine these results, our findings provide promising evidence that SCU may be a viable tool for OA therapeutic intervention.

## Data Availability Statement

All datasets generated for this study are included in the article/supplementary material.

## Ethics Statement

The animal study was reviewed and approved by The Animal Care and Use Committee of Wenzhou Medical University (ethics code: wydw2017-0159).

## Author Contributions

JX designed the experiments. ZL and ZH performed the experiments. JW and WN performed the supplementary experiments. XL, WS, and LS prepared figures and statistical analysis. ZL and YB wrote the original manuscript. ZH, SL, YS, and GZ edited the manuscript. The results and the ﬁnal manuscript were reviewed by all authors.

## Funding

This work was supported by grants from the Wenzhou Public Welfare Science and Technology Project (Y20170238), the National Natural Science Foundation of China (81401798), the Zhejiang Provincial Natural Science Foundation of China (LQ13C100001, LY17H060009), the Zhejiang Provincial Project for Medical and Health Science and Technology (2013KYA128), and the Wenzhou Municipal Science and Technology Bureau (Y20190078).

## Conflict of Interest

The authors declare that the research was conducted in the absence of any commercial or financial relationships that could be construed as a potential conflict of interest.
